# Continuous Flow Synthesis of Copper Oxide Nanoparticles Enabling Rapid Screening of Synthesis‐Structure‐Property Relationships

**DOI:** 10.1002/smll.202403529

**Published:** 2025-01-05

**Authors:** Matt Jellicoe, Yibo Yang, William Stokes, Matthew Simmons, Lina Yang, Stephanie Foster, Zabeada Aslam, Jennifer Cohen, Ashi Rashid, Andrew L. Nelson, Nikil Kapur, Rik Drummond‐Brydson, Thomas W. Chamberlain

**Affiliations:** ^1^ Institute of Process Research and Development School of Chemical and Process Engineering University of Leeds Woodhouse Lane Leeds LS2 9JT UK; ^2^ Institute of Process Research & Development School of Chemistry University of Leeds Woodhouse Lane Leeds LS2 9JT UK; ^3^ Institute of Process Research and Development School of Mechanical Engineering University of Leeds Woodhouse Lane Leeds LS2 9JT UK

**Keywords:** continuous flow, nanoparticles, optimization, toxicity

## Abstract

An adjustable and scalable method for the continuous flow synthesis of cupric oxide nanoparticles (CuO NPs), targetted the reduction of their activity to synthetic biomembranes to inform the fabrication of nanoparticles (NPs) with reduced toxicity for commercial applications. By manipulating key factors; temperature, residence time, and the ratio of precursor to reductant, precise control over the morphology of CuO NPs is achieved with X‐ray diffraction (XRD) and transmission electron microscopy (TEM) confirming the formation of needle‐shaped CuO NPs. One‐variable‐at‐a‐time studies reveal a relationship between the synthesis conditions and the characteristics of the resultant NPs, with CuO NPs varying controllably between 10–50 nanometres in length and 4–10 nanometres in width. Subsequently, Design of Experiment (DoE) exploration of the biomembrane activity of the CuO NPs intriguingly revealed only minimal effects on their membrane‐disruptive properties in the chemical space defined by the synthesis conditions explored. This study marks a significant milestone, as it introduces a facile, easy to scale, continuous flow synthesis of CuO NPs, with control over the length and width of the needle NPs and reveals that, regardless of the exact shape, the NPs have minimal impact on biomembranes, prompting more detailed exploration in the future for use in biomedical applications.

## Graphical abstract

This adjustable and scalable method for the continuous flow synthesis of cupric oxide nanoparticles (CuO NPs), targets reducing their toxicity. The facile, easy‐to‐scale, continuous flow synthesis of CuO NPs, with control over the length and width of the needle NPs, reveals that the nanoparticles have minimal impact on biomembranes, prompting more detailed exploration for use in biomedical applications.

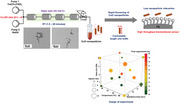

## 1 Introduction

Nanostructured metal oxides, which belong to a specific category of NPs, have garnered significant interest within the scientific community in recent years. This interest is attributed to the physical and chemical phenomena that arise due to quantum confinement at the nanoscale. Metal oxide NPs, such as iron oxide (Fe_3_O_4_) and titanium dioxide (TiO_2_), have found applications in drug delivery, biosensing, and cancer therapy due to their biocompatibility and magnetic properties.^[^ ^1^ ^]^ CuO NPs are one of a number of transition metal oxides that display size‐specific properties compared to their bulk and atomic/molecular counterparts. The narrowing of the band gap associated with the reduction in size from the bulk to the nanomaterial, the high solar absorbance, and the thermal conductivity of the nanomaterials have led to applications in biomedicine, sensing, energy storage, and catalysis.^[^ ^2^ ^]^ In comparison to other metal oxide NPs CuO NPs offer superior antimicrobial, anticancer, and drug delivery capabilities in biomedicine, along with high specific capacity, cycling stability, and cost‐effectiveness in energy storage applications like lithium‐ion batteries.^[^ ^2^ ^]^ In catalysis, their redox activity, visible light photocatalysis, and low cost make them efficient alternatives to other metal oxides and noble metal‐based catalysts for various reactions.^[^ ^2^ ^]^

Although CuO NPs offer significant advantages over other metal oxide NPs, their functional properties are highly dependent on morphology, with size and shape being strongly influenced by the synthetic method used.^[^ ^3^, ^4^ ^]^ Even slight modifications in reaction parameters can significantly impact the size and shape of the resultant NPs.^[^ ^3^, ^4^ ^]^ Thus, considerable emphasis has been placed on developing the capability to precisely adjust the reaction parameters to form NPs of the desired size and shape. To date, CuO NPs have been only synthesized commercially via batch processes, despite batch processing having low mixing and heat transfer within the reaction vessel resulting in batch‐to‐batch variability in sample morphology.^[^ ^5^ ^]^ This is remarkable, given that over the last 20 years, transfer to continuous flow synthesis has been widely reported to reduce such issues for other inorganic NPs.^[^ ^6^ ^]^ For example, gold and silver NPs have been synthesized using continuous flow techniques, with in‐line analysis employed to monitor and ensure the quality of the resultant products.^[^ ^7^, ^8^ ^]^ Semiconductors such as quantum dots (QD) have also undergone extensive study in flow and found pivotal applications in biosensing and bioimaging.^[^ ^9^, ^10^ ^]^ Metal oxides of elements such as iron, titanium, and zinc have also been synthesized under flow conditions, but their manipulation (control of size and shape) has proved challenging.^[^ ^6^, ^11^ ^]^

Cupric (CuO) and cuprous oxide (Cu_2_O) NPs have been synthesized in flow conditions previously at lab scale (**Table** **1**), Nikam et al. achieved the successful synthesis of CuO by seamlessly integrating microwave and flow synthesis techniques.^[^ ^12^ ^]^ They demonstrated the ability to modulate the shape and size of CuO NPs by adjusting the copper precursor concentration during the reaction. While this method is effective, it comes with a considerable cost, has limited scalability due to difficulties in controlling the reaction temperature via microwave heating, and lacks an underlying relationship between conditions and CuO NP size and shape. Separately, Al‐Antaki et al. synthesized Cu_2_O NPs through the laser irradiation of a copper rod in a continuous flow, vortex fluidic device.^[^ ^13^ ^]^ Subsequently, they transformed these Cu_2_O NPs into CuO NPs, by heating the as‐prepared Cu_2_O nanoparticle solution in air at 50 °C for 10 h. The resultant material has achieved high yields and with noteworthy precision (d_NP_ = 11 ± 1 nm). However, this approach relies on an expensive and intricate reactor system (a vortex fluidic device) and includes a batch, post‐processing step to convert Cu_2_O to CuO, making it difficult to scale beyond the research lab (maximum throughput predicted to be 200 mg in 12 h). In addition, there is no reported control of the size or shape of the CuO NPs. Consequently, there is an unmet need for a more straightforward, scalable, and efficient method for controllably producing CuO NPs of a predefined size within a continuous flow system. Moreover, we believe that it is important when designing a process with potential for large‐scale applications to consider the societal obligation of incorporating safe and sustainable design metrics to the process to limit exposure for production workers.^[^ ^14^, ^15^ ^]^

**Table 1:** A summary of the state and art in CuO synthesis is flow and comparison with this work.

Study	Method	Precursor	Material	Size and shape	Scalable	Tuneable	Cost	Limitations
Nikam et al^[^ ^11^ ^]^	Continuous flow in aqueous solution through a CSTR in a microwave oven (1 min residence time, 2.45 GHz, 700 W irradiation).	Cu(acetate)_2_	CuO NPs (70% yield)	Dependent on temp. and conc. of precursor; Nanobelts 14 × 120 nm, spherical NPs ≈4.5 nm	5 g h^−1^	Yes, but with no underlying understanding.	Medium	Scale up effects the temperature and thus the yield of NPs.
Rastom et al^[^ ^12^ ^]^	Continuous flow of aqueous solution through a vortex fluidic device with laser ablation of static Cu rod (15 min residence time), followed by thermal batch treatment (50 ⁰C in the air for 10 h)	Cu metal rod	Cu_2_O NPs (CuO NPs after thermal treatment) (yield not specified)	Cu_2_O NPs 19–14 ± 1–2 nm (CuO NPs 11 ± 1 nm)	200 mg in 12 h	No	High	Batch thermal treatment is required to convert to CuO NPs
This work	Continuous flow in ethanol solution through coiled, tubular flow reactor (50–100 ⁰C, 5–20 min residence time)	Cu(acetate)_2_	CuO NPs (87% yield)	Nanorods 9–50 × 3‐8 nm	Yes	Yes, size and aspect ratio are related to res. time, temp. and red. conc.	Low	

The toxicity of metal oxide NPs is a critical consideration when the intended use is in biomedical engineering applications, with samples having to exhibit non‐toxic properties toward mammalian cells.^[^ ^1^ ^]^ Previously this has been explored using a high‐throughput synthetic biological analysis system, called the Automated Biomembrane Screening Platform (ABSP), which has been developed to approximate nanomaterial‐induced damage to biological membranes.^[^ ^16^, ^17^ ^]^ This method is shown to correlate well with standard cell viability studies for peak suppressions above 20%, but offers a significant advantage over other, multi‐well plate‐based, nanoparticle toxicity assessment techniques in terms of speed of data acquisition.^[^ ^16^, ^17^ ^]^ In addition, as the phospholipid monolayer can be quickly re‐established the electrode is reusable, enabling high‐throughput screening and eventually integration for in‐line measurements. In this work, we present a method for synthesizing and controlling the morphology of the CuO needle NPs in a continuous flow setup, targeting both size and shape control and simultaneously minimizing any adverse effects the NPs have on synthetic biomembranes. Subsequently, we employ an innovative artificial biomembrane sensor to assess the impact of the CuO NPs on biological membranes.

## 2 Results and Discussion

**Figure 1:**
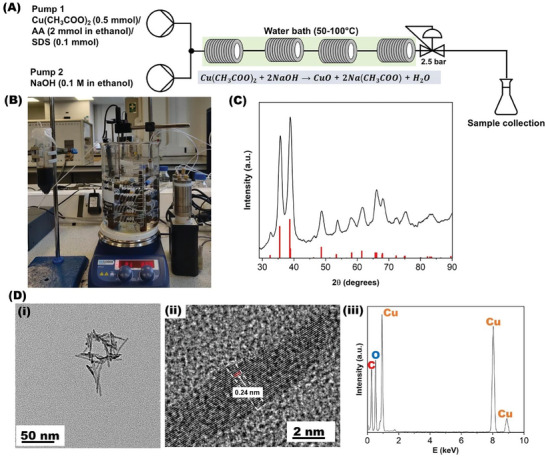
A) Schematic diagram of the continuous flow reactor showing four linked coiled flow inverter reactors (CFIRs) for the synthesis of CuO NPs. B) A photograph of the continuous flow rig. C) XRD of the NPs synthesized under continuous flow with the following reaction parameters: 78 °C, a 5‐min residence time and a precursor‐to‐reductant ratio of 1:1 (CuO ICDD reference 04‐007‐0518, red lines). D) Representative bright field TEM image of the CuO NPs synthesized under continuous flow showing: (i), the particle size of the CuO NPs (ii), a higher magnification TEM phase contrast image of an individual single crystal needle exhibiting a lattice fringe spacing of 0.238 nm corresponding to either the (111) or (200) spacings of CuO (0.232 and 0.230 nm respectively), and (iii) an energy dispersive X‐ray (EDX) spectrum of the CuO NPs (Figure S22, Supporting Information for EDX maps).

The bulk synthesis procedure, employed by Kida et al., in which copper acetate is mixed with sodium hydroxide and precipitated in ethanol at 78 °C.^[^ ^18^ ^]^Our synthesis employs a coiled flow inverter reactors (CFIRs), a reactor with initial mixing provided a T‐piece, as illustrated in **Figure** **1**a,b, wherein four microreactors are interconnected to enable appropriate reaction times, see Supporting Information for full details of the reactor system. CFIRs have been used previously to minimize the coalescence and agglomeration of silver NPs due to the laminar flow regime in the microreactor. Mixing was maintained through the length of the reactor through the use of a previously reported, helix‐wound tubular reactor, to induce Dean vortices within the flow.^[^ ^19^ ^]^ This is further enhanced by periodically rotating the direction of the secondary flow through switching the direction of the helical coil, which further reduces the residence time distribution.^[^ ^20^ ^]^The precipitation from alcohol allows the direct formation of CuO, in contrast to precipitation from an aqueous solution, which results in the formation of Cu(OH)_2_.^[^ ^18^ ^]^To transfer the synthesis to flow, the ratio of the copper acetate to the sodium hydroxide was initially kept constant to avoid altering the chemistry and stability of the CuO NPs.

TEM allowed us to observe the needle‐like morphology of the CuO NPs and EDX analysis showed the uniform distribution of copper to oxygen with an atomic ratio of 1:1 (O: 12.7 and Cu: 50.8 wt.%) with carbon from the support film also present in the EDX, Figure 1d‐iii, consistent with previously reported data for CuO NPs by Nagashree et al.^[^ ^21^ ^]^ High‐resolution TEM of individual needles revealed a lattice fringe spacing of 0.238 nm indicating the short needle axis was most likely along <111>, Figure 1d‐ii, Figures S20 and S21 (Supporting Information).^[^ ^22^ ^]^ Analysis of the X‐ray diffraction (XRD) pattern of the product synthesized at 78 °C, 5 min residence time, and a precursor‐to‐reductant ratio of 1:1 shows good agreement with the ICDD reference for Tenorite CuO (04‐007‐0518), suggesting phase purity and crystallite size of ≈9 nm based on Scherrer broadening of the hkl peaks, which corresponds to the average needle width, see Table S1 (Supporting Information).

**Figure 2:**
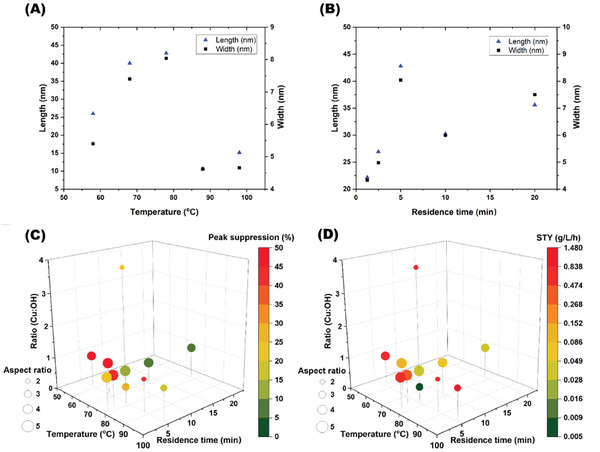
A) Graph showing the effect of temperature on the length and width of the CuO NPs. B) Graph showing the effect of residence time on the length and width of the CuO NPs (error bars less than 1.2 nm). C,D) One factor at a time experiments (OFAT) showing: the effect of conditions on the aspect ratio and peak suppression in the artificial biomembrane, and the aspect ratio and space‐time yield (STY) of the CuO NPs.

Transitioning to flow enabled us to rapidly explore and determine the chemical space within which the CuO NPs can be formed. A one‐factor at a time (OFAT) method was employed to find the relationship between the specific conditions: temperature, residence time, and the ratio of copper precursor to NaOH, and the synthesized CuO NPs, **Figure** **2** (Table S1 and Figures S9–S11, Supporting Information).^[^ ^23^ ^]^The first parameter explored was the effect of temperature as a number of studies in batch have shown that temperature is one of the key factors in controlling the size and shape of the CuO NPs.^[^ ^24^, ^25^, ^26^ ^]^ While batch synthesis was undertaken at 78 °C, the boiling point of ethanol, under flow conditions, the back pressure regulator in the system enabled elevated temperatures to be used to increase the rate of the reaction. Multiple reaction temperatures were investigated (58–98 °C) at a residence time of 5 min, Figure 2a. From TEM observations, the formation of CuO NPs with low variability of < ±1.7 nm in length and < ±1.1 nm in width is observed in all samples, Tables S1 (Supporting Information). Raising the temperature from 58 to 78 °C increased the length and width of the needles, while a further increase in temperature resulted in a significant decrease (reduced by 70%) in both the length and width of the needles.

Subsequently, the residence time of the reaction was investigated and varied from 1.25 to 20 min. Overall, a general trend of longer residence time leading to increased length and width of the CuO NPs was observed, Figure 2b and Table S1 (Supporting Information). The lowest residence time 1.25 min produced noticeably short and narrow NPs (22 and 4 nm, respectively). Another parameter investigated was the ratio of the copper precursor to sodium hydroxide. Zheng et al. reported that a ratio of 2:1 was essential in batch as deviation from this ratio resulted in incomplete reactions.^[^ ^18^ ^]^In our continuous synthesis, altering the ratio of the precursor to reductant significantly from 2:1 (we explored from 0.5 to 4) generally led to a significant decrease in the length and width of the NPs produced, Table S1 (Supporting Information), but no decrease in conversion. Interestingly, a 1:1 ratio of the copper precursor to sodium hydroxide (Cu:OH) seems to produce the largest CuO needles, with a length and width of 42 and 8 nm respectively (full conditions, residence time of 5 min, at 78 °C and a 1:1 ratio (Cu:OH)). Increasing the Cu precursor to reductant ratio has been shown to decrease the size and increase the yield of CuO nanoflowers.^[^ ^27^ ^]^ Moreover, a decrease in Cu precursor concentration has been shown to decrease the size of CuO NPs.^[^ ^28^ ^]^ Given the low density and linearity of the data in our OFAT exploration of the multi‐dimensional chemical space, care must be taken not to over interpret the relationships between reaction conditions and the resultant CuO NPs. In the future, the integration of on line analytics for nanoparticle sizing (e.g., DLS, SAXS, or XRD) and complex, dynamic ramping of input parameters would enable more detailed, data‐dense experimentation, which is required to explore the precise nature of the relationships between the reaction variables and the resultant CuO NPs more definitively and underpin a knowledge‐based model.

**Figure 3:**
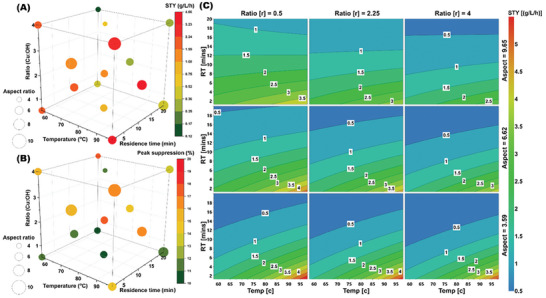
A) Design of experiment (DoE) results showing the effect of the conditions on aspect ratio and STY, and B) the peak suppression and the aspect ratio. C) a 4D contour plot showing the effect of the conditions on the space‐time yield.

Following the OFAT testing, a design of experiments (DoE) methodology was employed to further explore the system, specially exploring temperature, residence time, precursor‐to‐reductant ratio within the limits determined to be influential on the CuO NP formed from OFAT data, and the physical limits of the reactor platform, Table S2 (Supporting Information) for full details. DoE is a highly effective and widely employed optimization technique, especially in the pharmaceutical and fine chemical sectors, and enabled us to simultaneously find the optimal conditions for efficient processing, space‐time yield (STY, which factors in the amount of material produced relative to the time and the volume of liquid required) and control the nanoparticle morphology (aspect ratio) **Figure** **3** and Figures S12–S15 (Supporting Information).^[^ ^23^, ^29^, ^30^, ^31^, ^32^ ^]^ Yields varied from 4% to 87%, depending on conditions, Table S2 (Supporting Information) for full details. We observed that short residence times (1.25 min) and low temperatures (58 °C) led to high aspect ratio NPs Figure 3c. In our case, we choose to visualize the space‐time yield (STY), and aspect ratio of the CuO NPs, Figure 3a. We found that at higher temperatures, high precursor‐to‐reductant ratios and low residence times led to the synthesis of CuO NPs 28 nm in length and 4.7 nm in width (5.98 aspect ratio) with a high STY, Table S2 (Supporting Information). This allowed us to both fit a model to predict the scale‐up of nanoparticle size and find the optimal conditions in terms of STY, Figure S16 (Supporting Information). The response contour showed that residence time and temperature were the most influential factors being positively correlated with the STY. While a higher precursor‐to‐reductant ratio resulted in a significant but less pronounced increase in STY.

Using a previously reported artificial biomembrane sensor platform (ABSP) off‐line, we were able to identify the membrane disruption of the CuO NPs produced under continuous flow.^[^ ^16^ ^]^ The CuO NPs synthesized in the OFAT were investigated to determine the effect of the reaction conditions (temperature, residence time, and the ratio of Cu:OH) on the observed peak suppression of the resultant CuO NPs, Figure 2c (Table S1, Supporting Information for biomembrane analysis). We observed via control experiments that the starting materials had no impact on the biomembrane sensor, Figure S25 (Supporting Information). Each condition modulated during the OFAT experimentation influenced the membrane disruption of the resultant NPs to varying degrees (5–50% suppression). This is expected as the toxicity of CuO is well documented, with the key factors that influence the toxicity of CuO NPs including, particle shape, size, surface functionalization, and time–dose interaction explored for animal and cell models.^[^ ^33^ ^]^Cu(II) forms strong complexes with organic ligands as it is next to Hg(II) in the Irving–Williams order,^[^ ^34^ ^]^ and this is how it exerts its toxicity, i.e., forms strong bonds with enzymes, etc.^[^ ^35^ ^]^ So in the ABSP Cu(II) will bind to the negative phosphate group of lecithin (DOPC) in the artificial membrane which would disrupt its structure. Therefore, we postulate that the small and quantifiable toxicity observed in all samples is a result of reactive metal ions on the surface of the CuO NPs, in line with previous reports.^[^ ^36^, ^37^, ^38^ ^]^

However, we observed that there was no significant relationship between nanoparticle morphology and the resultant peak suppression, Figure S23 (Supporting Information), with no distinguishable relationship between the space‐time yield and the peak suppression observed in the ABSP, Figure 2c,d. The OFAT experiments identified a region that provided a good balance between productivity (a high STY) and producing NPs with low membrane disruption, at a residence time of 5 min, the ratio of 1:1 (copper precursor to NaOH) and 98 °C, Figure 2c. This corresponds to a peak suppression of 17%, along with a high STY (1.48 g L^−1^ h^−1^), Figure 2c,d.

Exploring the relationship between NP synthesis efficiency (STY), the resultant structure (morphology), and resultant biomembrane disruption, using a more focused DoE identified a productive region of chemical space in which low membrane disruption with peak suppression of < 20% in all conditions investigated (Figure 3a,b; Table S2, Supporting Information). There was a moderate relationship (r^2^ 0.3–0.5) between increased CuO needle length and width and an increase in peak suppression, Figure S24 (Supporting Information). While no relationship was found between the aspect ratio or STY and the peak suppression observed in the ASBP.

## 3 Conclusion

In summary, our study has demonstrated the first continuous flow synthesis of CuO NPs using a newly developed microfluidic device consisting of multiple coiled flow inverter reactors. We demonstrate that the morphology of these CuO NPs can be precisely controlled by adjusting key parameters such as temperature, residence time, and the precursor‐to‐reductant ratio. The resulting CuO NPs range in size from 10 to 50 nanometres in length and 4 to 10 nanometres in width. Furthermore, exploration of the membrane‐disruptive properties showed that CuO NPs have a small and quantifiable disruptive effect on artificial biomembranes under various conditions. This research presents an innovative, scalable, controllable, safe, and sustainable design approach to continuous flow synthesis of CuO NPs with tailored morphology, offering new possibilities for applications in various fields, including cancer treatment.^[^ ^39^ ^]^ Excitingly, moving forward, the translation of this synthesis to flow will now enable the implementation of online monitoring of both nanoparticle structure (DLS, SAXS etc.) and properties, including toxicity (biomembrane disruption measurements) and optical properties (fluorescence, absorbance etc.), to be combined with self‐optimization algorithm based dynamic control, to enable informed process development and consistent product quality, which will dramatically enhance the utilization of base metal oxide NPs for specific applications.

## Conflict of Interest

The authors declare no conflict of interest.

## Author Contributions

M.J. led the writing of the paper and analysis of the results, Y.Y. undertook most of the experiments, L.Y. undertook some experiments and analysis, and W.S. and A.R. undertook the artificial biomembrane experiments. M.S. M.J. and N.K. designed, developed, and modified the microfluidic device. S.F. and Z.A. conducted the TEM experiments and imaging. A.L.N. and W.S. designed and developed the artificial biomembrane sensor. M.J. R.D.B. and T.W.C. drafted the manuscript. All authors contributed to the writing, editing, and publishing of the paper.

## Supporting information



Supporting Information

## Data Availability

The data that support the findings of this study are available from the corresponding author upon reasonable request.
