# Supplementary material for: Continuous Flow Synthesis of Copper Oxide Nanoparticles Enabling Rapid Screening of Synthesis‐Structure‐Property Relationships
Source: Small. 2025 Jan 5;21(6):2403529. doi: 10.1002/smll.202403529 (PMC11817908; doi:10.1002/smll.202403529)
Supplement: Supplementary file 1 — Supporting Information [file SMLL-21-2403529-s001.docx]

Supplementary information

**Continuous Flow Synthesis of Copper Oxide Nanoparticles Enabling Rapid Screening of Synthesis-Structure-Property Relationships**

Matt Jellicoe^1,2^*, Yibo Yang^1^, William Stokes^2^, Matthew Simmons^1^, Lina Yang^1^, Stephanie Foster^1,2^, Zabeada Aslam^2^, Jennifer Cohen^2^, Ashi Rashid^2^, Andrew L. Nelson^2^, Nikil Kapur^3^, Rik Drummond-Brydson^1^, and Thomas W. Chamberlain^2^*

1 Institute of Process Research & Development, School of Chemical and Process Engineering, University of Leeds, Woodhouse Lane, Leeds, LS2 9JT UK.

2 Institute of Process Research & Development, School of Chemistry, University of Leeds, Woodhouse Lane, Leeds, LS2 9JT UK.

3 Institute of Process Research & Development, School of Mechanical engineering, University of Leeds, Woodhouse Lane, Leeds, LS2 9JT UK.

**Methods**

**Chemicals**

Unless otherwise stated, chemicals were obtained from Sigma Aldrich. Cu(CH_3_COO)_2_ (CAS:142-71-2), acetic acid (CAS: 64-19-7), sodium dodecyl sulfate (≥99.0%, CAS: 151-21-3), and sodium hydroxide (≥98.0%, CAS: 1310-73-2). All water used was ultrapure (~14 MΩ, Purite).

**Precursors for CuO synthesis**

Cu(CH_3_COO)_2_ (CA, 0.5 mmol), acetic acid (AA, 0.025 mmol), and sodium dodecyl sulfate (SDS, 0.1 mmol) were added to ethanol (60 mL) and stirred for 10 minutes. NaOH (0.4 mmol) was dissolved in ethanol (40 mL). Pump A feed line was added to the CA/AA/SDS solution and pump B feed line was added to the NaOH solution.

**Construction of continuous flow reactor for CuO synthesis**

Three continuous flow inverted reactors were 3D printed from Digital ABS resin.^1^ Tolerances on these prints are approximately 0.1 mm with layer heights of approx. 16 microns. The 3D printer technicians clean the components post printing with a high-powered water jet; however, it is useful to sonicate these in washing up liquid for 20 minutes after this to remove any debris. After cleaning of the 3D printed CFIRs these require heat treatment to increase layer bonding and increase tensile strength – helping to prevent any structural failures when used in the system. Each CFIRs were heated to 80°C for 12 hours before cooling to room temperature. A second round of heating was then used to heat the pieces to 100°C for 12 hours before cooling to room temperature. An optional third heating stage can then be used to heat these to 120°C for 12 hours before cooling to room temperature. To set up the heating stage 1 x Growth Stage Support, 1x PFA Tubing PFA Tubing (I.D. 1.5 mm, O.D.) and 2 x PFA Tubing (I.D. 1 mm, O.D.) (Figure S1a). Place the end of the I.D. 1.5 mm through one of the side holes on the Growth Support Stage, leaving approx. 5 cm sticking out of the opposite side. Wrap this around the nearest spiral following this as it moves around the arm (Figure S1b).


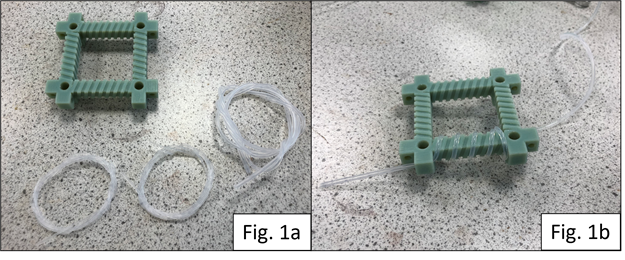


**Figure S1.** Photos of the reactor tubing on the 3D scaffold.

Continue to wrap the PFA Tubing (I.D. 1.5 mm, O.D.) around the remaining arms following each spiral, moving onto alternating spirals on each arm. To help visualize this process the alternating spirals have been colour coded red and blue in Figure S2a. Once all spirals have been completed thread the remaining tube through the side hole next to where the tubing was initially threaded. Take the 2x PFA Tubing (I.D. 1.0 mm, O.D.), thread these into the side holes on the opposite corner of the support to those already threaded. Wrap the tubing around the remaining spiral on the support not already occupied by the PFA Tubing (I.D. 1.5 mm, O.D.) tubing. Threading these through the side hole next to where the tubing was initially threaded (Figure S2b).


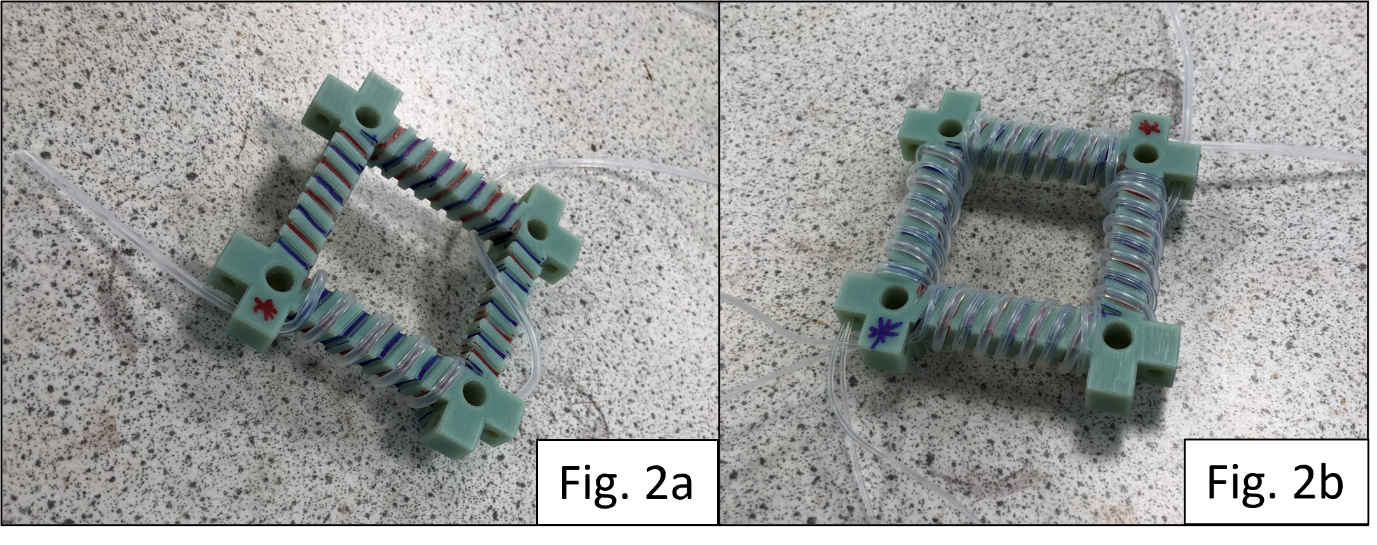


**Figure S2.** Photos of the reactor tubing on the 3D scaffold.

Repeat the above process for the remaining two growth stages, wrapping 2x PFA Tubing (I.D. 1.5 mm, O.D.), around the spirals of each. After this has been completed you should have, 3 growth stage supports, 1 which has 1 x PFA Tubing (I.D. 1.5 mm, O.D.) and 2 x PFA Tubing (I.D. 1.5 mm, O.D.) wrapped around it in alternating spirals and, 2 x growth stage supports which have 2x PFA Tubing (I.D. 1.5 mm, O.D.) wrapped around them (Figure S3.).


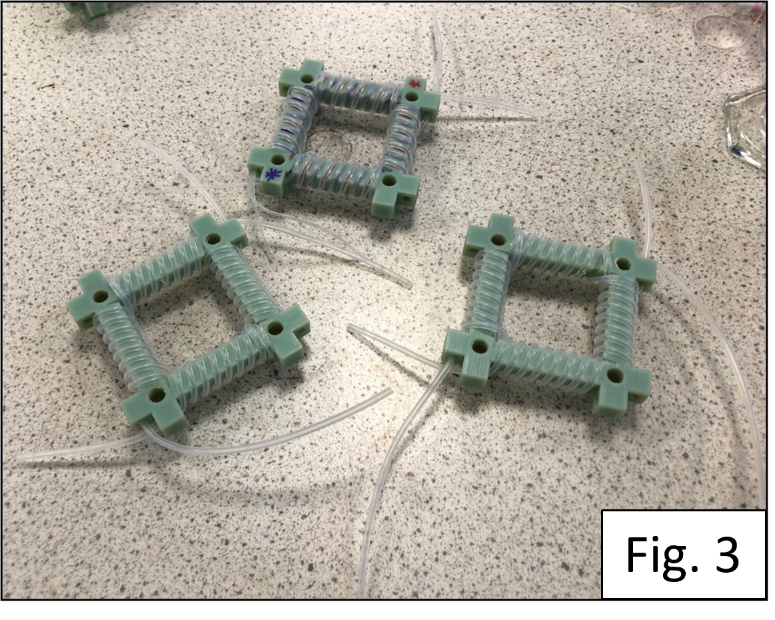


**Figure S3.** Photo of the reactor tubing on the 3D scaffolds.

Take the 1 of the bolts and 1 of the growth stage supports which has been prepared with 2x PFA Tubing (I.D. 1.5 mm, O.D.) tubing. Screw the bolt through one of the holes through the support legs, pushing this through until it is flush with the growth stage support (Figure S4a). Take the second growth stage prepared with 2x PFA Tubing (I.D. 1.5 mm, O.D.) tubing, and screw this onto the bolt placed through the previous growth stage, until this is flush with the growth stage below (Figure S4b). Finally take the growth stage support containing the heating stage tubing (2x PFA Tubing (I.D. 1.0 mm, O.D.)) and nucleation stage (1x PFA Tubing (I.D. 1.5 mm, O.D.)) and screw this onto the bolt. There should now be a structure with a growth stage support containing 2x PFA Tubing (I.D. 1.5 mm, O.D.) tubing on the bottom and in the middle with a growth stage support containing the heating stage tubing (2x PFA Tubing (I.D. 1.0 mm, O.D.)) and nucleation stage (1x PFA Tubing (I.D. 1.5 mm, O.D.)) on top, (Figure S4c).


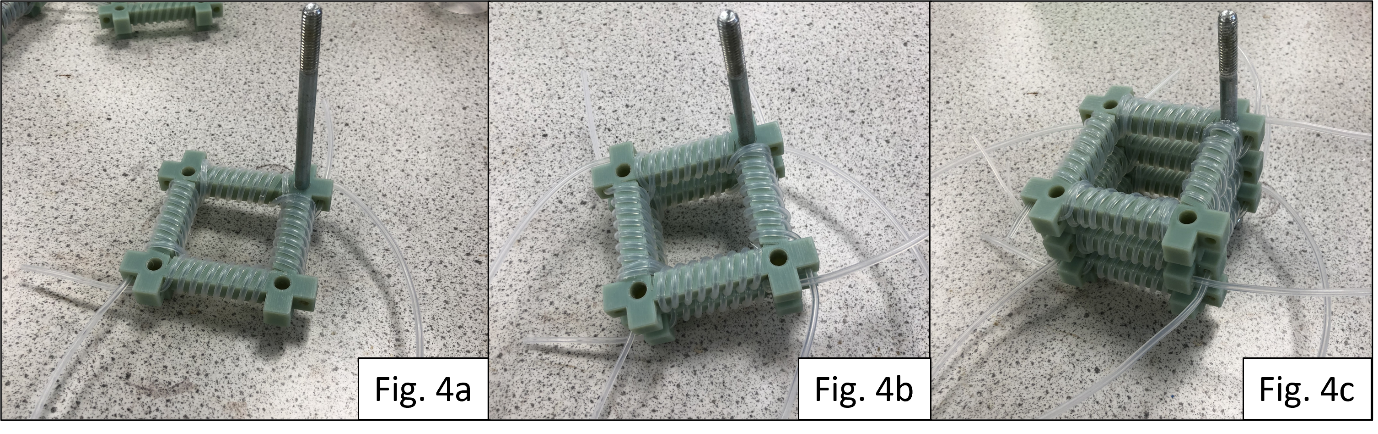


**Figure S4.** Photos of the reactor tubing on the 3D scaffold.

Take the remaining 3 bolts and screw these into and push them through the leg holes and push this flush with the bottom growth stage support (Figure S5).


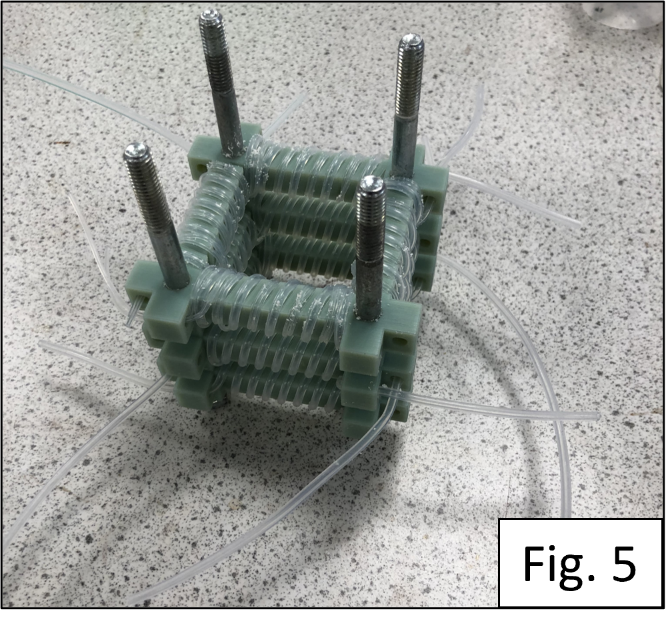


**Figure S5.** Photo of the reactor tubing on the 3D scaffold.

Take the PEEK ferruless fittings for the PFA Tubing (I.D. 1.0 mm, O.D.), and connect these to one end of each piece PFA Tubing (I.D. 1.0 mm, O.D.), by first threading the PEEK ¼”-28 fitting onto the tubing before pushing on the blue connector (Figure S6a). For the other two ends take the PTFE ferruleless fittings for the PFA Tubing (I.D. 1.0 mm, O.D.) Repeat for all four ends of the PFA Tubing (I.D. 1.0 mm, O.D.) (Figure S6b).


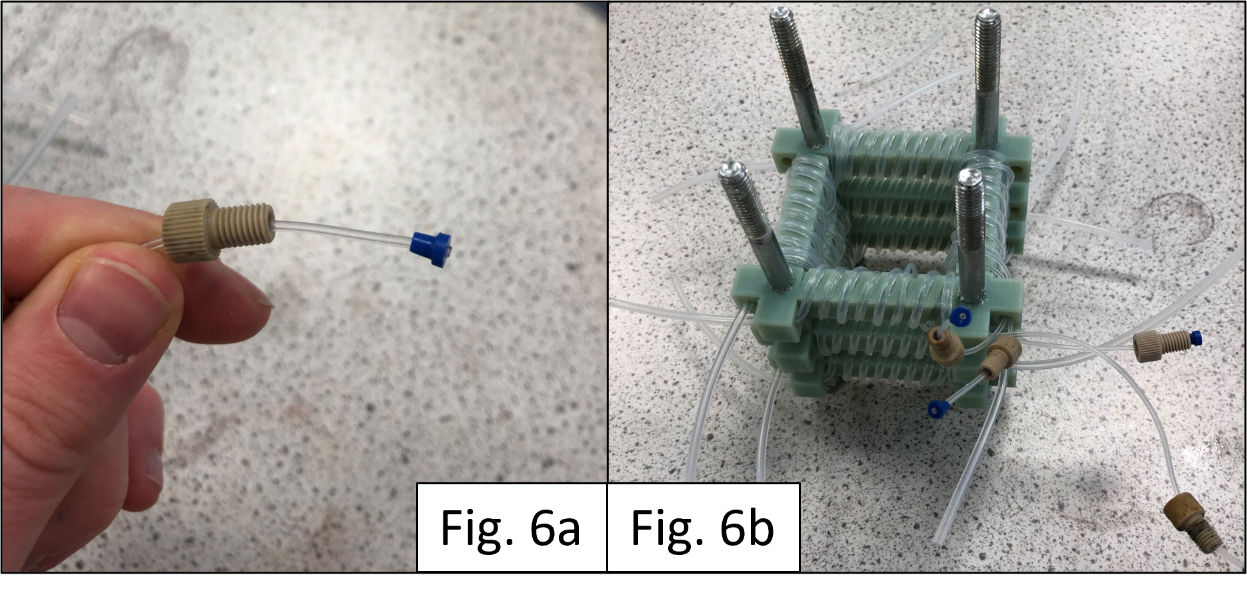


**Figure S6.** Photos of the connectors used for the reactor tubing.

Take the 8x PEEK ferruless fittings for the PFA Tubing (I.D. 1.5 mm, O.D.), and connect these to one end of each piece end of the PFA Tubing (I.D. 1.5 mm, O.D.) on the bottom two growth stages, by first threading the PEEK ¼”-28 fitting onto the tubing before pushing on the yellow connector. For the PFA Tubing (I.D. 1.5 mm, O.D.) on the top stage, take 1x PEEK ferruless fittings for the PFA Tubing (I.D. 1.5 mm, O.D.) and connect this to one end of the tubing. To the other take a PTFE ferruless fittings for the PFA Tubing (I.D. 1.5 mm, O.D.) and place this on this end. All tubing ends should now have a ferrueless fitting placed upon (Figure S7).


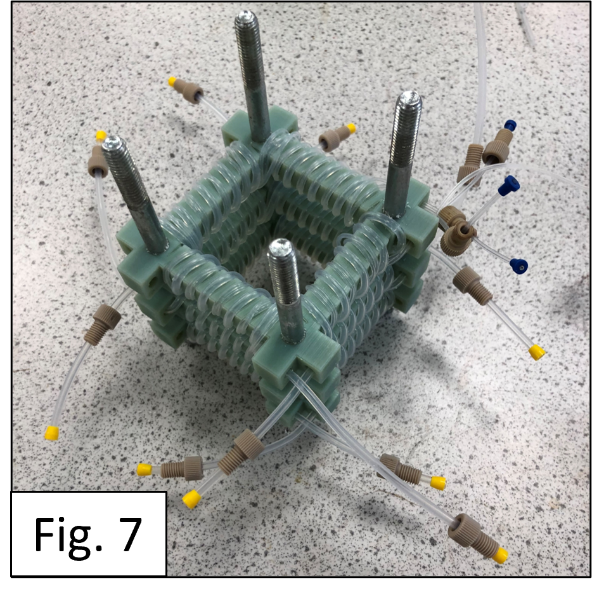


**Figure S7.** Photo of the reactor tubing on the 3D printed scaffold.

Take the T-piece with the threads for the 1/4”-28 tooled into the inlets. To the single tooled inlet connect the 1x PTFE ferrule of the PFA Tubing (I.D. 1.5 mm, O.D.) of the nucleation stage and connect this to the T-piece mixer, tightening it until the connector is flush and there is no movement of the tubing within the T-piece. Take the 2x PTFE connectors connected to the heating stage tubing PFA Tubing (I.D. 1.0 mm, O.D.), connect these into the T-piece where these are two inlets. Again, secure these so that they are flat against the T-piece and there is no give in the tubing. Take a single PEEK two-way connector, screw one end of the T-piece onto the connector for the tubing of the nucleation stage. Connect the opposite end of this to one of the tubing connectors for the first growth stage and repeat until all the stages are connected. A Back Pressure Regulator (BPR) was connected to the final ¼”-28 fitting for the final stage with PFA Tubing (I.D. 1 mm, O.D.) to collect the sample in a centrifuge tube (see fig 1b in main article).

**Continuous flow CuO synthesis**

The initial synthesis of CuO nanoparticles had the Milligat HF pumps running at 2 mL/min^-1^ and 1 mL/min^-1^ with mixed the Cu precursor with the reducing agent. The solution enters the CFIRs which are heated in a water bath at 78°C. During the experiment, black particles were observed forming within the reactor tube. The residence time was controlled at 5 minutes, and a 25 mL product sample was collected. The product was centrifuged washed 5 times with a 1:1 water/ethanol to remove the SDS, then a final water wash to remove the ethanol and the solution was re-dispersed in 10 mL of milliQ water. The solution was kept for off-line analysis unless a dry produced was needed and then we dried the solution in an oven over night at ~82°C. Reaction yields were calculated weighing the residual powdered after drying and ranged from 4-87%.

**Off-line analysis**

Samples for TEM were prepared by dropping a dilute suspension of the nanoparticles onto a grid and allowing the solution to evaporate. The grids were composed of a Formvar/carbon thin film on an Au mesh (Agar Scientific). The images were taken on a FEI Tecnai TF20: FEGTEM Field emission gun TEM/STEM operated at 200 kV and fitted with a HAADF detector, Oxford Instruments INCA 350 EDX system/80 mm X-Max SDD detector and Gatan Orius SC600A CCD camera. Measurements of particle size were analysed from >50 nanoparticles. The SAED pattern was taken on a TESCAN TENSOR dedicated scanning TEM operated at 100 kV, with a probe current of 400 pA.

The dried samples for XRD were prepared by placing a small amount of CuO nanoparticles on a XRD holder. A 30 minute scan was performed in normal Gonio (Bragg-Brentano) mode to obtain a diffractogram with a scan range of 10-80° 2θ and a step size of 0.013° for each sample .

**Off-line artificial biomembrane analysis**

A novel membrane-on-chip screening module (1) is coupled with electrical and fluidic components in an automated biomembrane screening platform (ABSP) which ensures control over reagent flow and allows for electrochemical interrogation. Membrane damage following interaction with a biomembrane-active species is detected by capacitance-current peak changes during the electrochemical interrogation of an Hg-supported phospholipid membrane monolayer, contained within the module itself. A strong correlation has been demonstrated between biomembrane interaction measured using this technique and a biological bilayer membrane (2).

Figure S8 displays a schematic of the membrane-on-chip screening technique to evaluate biomembrane interaction of NM, with peak ‘A’ highlighting the capacitance-current peak on a rapid cyclic voltammetry (RCV) trace, used during the quantitative comparison (1).
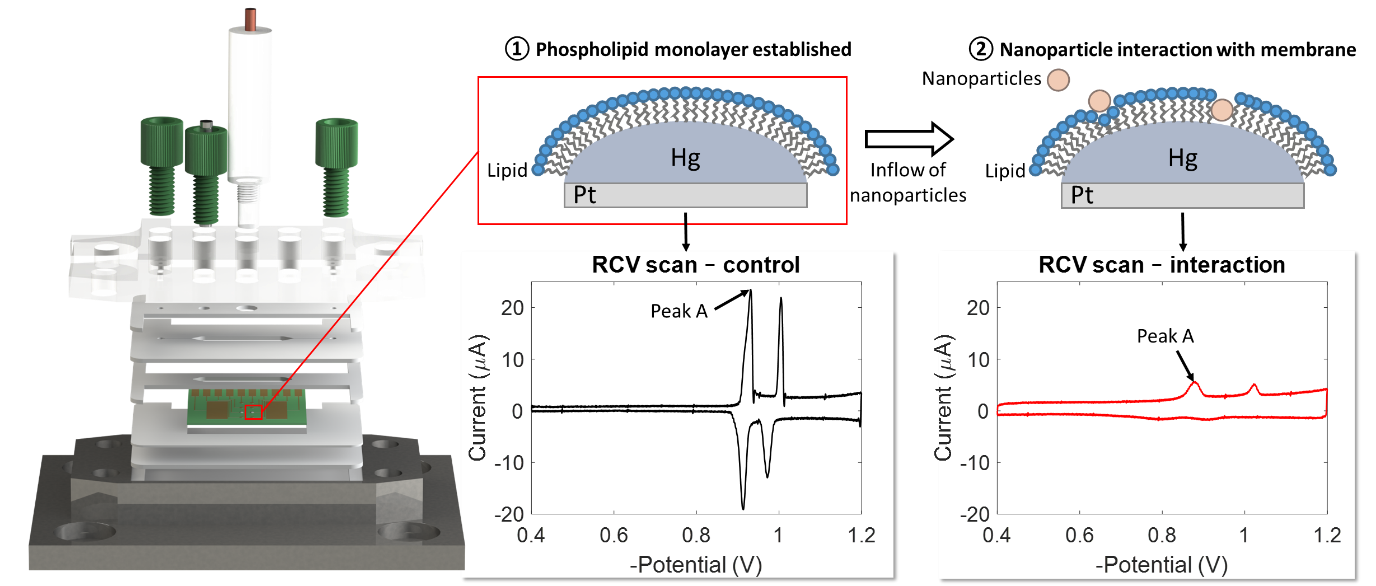


**Figure S8.** Schematic of the screening technique to evaluate biomembrane interaction of the nanomaterial displaying ***left:*** exploded assembly of the membrane-on-chip screening module and ***right:*** analysis of the Hg-supported phospholipid membrane monolayer through RCV response, with peak “A” highlighting the capacitance–current peak used to provide a quantitative comparison of biomembrane interaction.

To prepare the microfabricated electrode, prior to insertion in the flow cell, it was cleaned in 1 M NaOH/CH_3_OH base solution for approximately 12 hours, rinsed with Milli-Q water, neutralised with 18 M HCl, rinsed with Milli-Q water again and then dried. After cleaning, Hg was deposited onto two Pt bases prior to mounting in the flow cell. Once contained within the flow cell, an RCV potential excursion from −0.4 V to −3.0 V at a scan rate of 100 V s^−1^ was completed, for approximately 30 min, maintained under PBS throughout this period under static conditions to ensure robust adhesion of Hg to the Pt substrate was achieved and any organic material from the Hg surface was removed.

The fluids used during the screening procedure (PBS, DOPC, and sample) were prepared prior to testing by bubbling with argon gas, for a minimum period of 30 min prior to screening, to exclude dissolved oxygen (O2) in the fluids. After excluding O2, individual syringes were filled, mounted on the platform, and connected to the tubing. RCV scans were completed by applying potential excursions between two potentials at a specified scan rate, depending on the stage of the screening procedure. The four stages, controlled from the user interface as shown in the supplementary material, consisted of an “Idle,” “Clean,” “Lipid,” and “Sample” stage.

**Idle response:** During the “Idle” stage, a potential excursion from −0.4 V to −1.2 V was completed at a scan rate of 40 V s^-1^ under static conditions so that the RCV response could be analysed after each stage of the process. No fluid was flushed into the flow cell during this stage to allow for analysis of the RCV response. An appropriate control RCV response, prior to sample injection, could be confirmed by the operator and the resulting change in the RCV response after the sample was injected could be analysed. Data were exported from the “Idle” stage, where RCV responses were exported for further analysis.

**Flow cell clean:** During the “Clean” stage, a potential excursion of −0.4 V to −3.0 V was completed at a scan rate of 100 V s^-1^ under PBS flow at a constant flow rate of 4.0 cm^3^ min^-1^ to flush any remaining sample (and other contaminants) out of the flow cell into a waste container. A minimum of 5 cm^3^ of buffer was used to clean the flow cell after each measurement.

**Supporting a phospholipid membrane monolayer on Hg:** Once cleaned, DOPC was flushed into the flow cell at a flow rate of 1.0 cm^3^ min^-1^ in the “Lipid” stage. A potential excursion of −0.4 V to −3.0 V was completed at a scan rate of 100 V s^-1^ under PBS flow at a constant flow rate of 4.0 cm^3^ min^-1^, before returning to the “Idle” stage to assess the RCV response. A total of 0.5 cm^3^ of DOPC was injected to create a stable monolayer on the Hg electrode, confirmed through analysis of the RCV response showing the characteristic peaks, specific to the phospholipid in use, at appropriate potentials.

**Sample interaction with the phospholipid membrane monolayer:** To assess interaction with the DOPC layer, a test sample was flushed in during the “Sample” stage at a flow rate of 1.0 cm^3^ min^-1^ with PBS at a flow rate of 4.0 cm^3^ min^-1^. A potential excursion from −0.4 V to −1.2 V was completed at a scan rate of 40 V s^-1^, similarly to the “Idle” stage, to observe how the peaks changed in real-time during flow of the sample through the flow cell. All sample concentrations were screened three times by evaluating samples in the order of increasing concentration, starting with the lowest concentration. Another three repeats were completed by screening samples in a random order, to ensure that the results were not influenced by the order in which sample concentrations were screened. After completing a measurement, the syringe containing the test sample was cleaned with MilliQ water (or replaced if a significant interaction was observed). A total of 2 cm^3^ of PBS was also injected through the tubing connected to the sample syringe to flush any remaining sample through the tubing into the waste container. The PBS, DOPC, and sample syringes were replaced after all sample concentrations were screened, prior to completing repeat measurements. The flow cell was then cleaned by selecting the “Clean” process on the user interface to remove any remaining DOPC on the Pt/Hg electrode and flush out the sample, re-establishing the Pt/Hg electrode as a clean sensor available for the next measurement.

**Supplementary data**

**Table S1**: Table of the experimental conditions in the one factor at time (OFAT) and the corresponding responses.

| Temperature (Celsius) | Residence time (minutes) | Ratio (Cu:OH) | Length (nm) | Width (nm) | Peak suppression (%) | STY (g/L/h) | Aspect ratio (L/W) |
| --- | --- | --- | --- | --- | --- | --- | --- |
| 58 | 5 | 1 | 26.0±0.6 | 5.4±0.1 | 49 | 0.53 | 4.81 |
| 68 | 5 | 1 | 40.0±0.9 | 7.4±0.1 | 45 | 0.13 | 5.40 |
| 88 | 5 | 1 | 10.7±0.2 | 4.6±0.5 | 40 | 0.74 | 2.31 |
| 98 | 5 | 1 | 15.1±0.4 | 4.7±0.7 | 17 | 1.48 | 3.24 |
| 78 | 5 | 1 | 42.8±0.7 | 8.0±0.3 | 10 | 0.03 | 5.32 |
| 78 | 1.25 | 1 | 22.2±0.4 | 4.3±0.1 | 20 | 0.52 | 5.12 |
| 78 | 2.5 | 1 | 26.9±0.6 | 5.0±0.1 | 40 | 0.30 | 5.40 |
| 78 | 10 | 1 | 30.2±1.2 | 6.0±0.8 | 5 | 0.05 | 5.03 |
| 78 | 20 | 1 | 35.6±1.7 | 7.5±1.1 | 5 | 0.04 | 4.74 |
| 78 | 5 | 4 | 9.9±0.2 | 4.2±0.1 | 22 | 0.53 | 2.38 |
| 78 | 5 | 0.5 | 14.1±0.3 | 3.6±0.9 | 25 | 0.01 | 3.90 |

**Table S2**: Table of the experimental conditions in the central composite face centred (CCF) and the corresponding responses.

| Temp. (Celsius) | Res. time (minutes) | Ratio (Cu:OH) | Length (nm) | Width (nm) | Aspect ratio | Peak suppression (%) | STY (g/L/h) | Yield  (%) |
| --- | --- | --- | --- | --- | --- | --- | --- | --- |
| 58 | 1.25 | 0.5 | 27.0 | 5.0 | 5.4 | 11.65 | 1.97 | 41.1 |
| 98 | 1.25 | 0.5 | 28.5 | 4.7 | 6.0 | 14.16 | 4.65 | 87.3 |
| 58 | 20 | 0.5 | 28.0 | 4.5 | 6.2 | 10.56 | 0.51 | 16.8 |
| 98 | 20 | 0.5 | 38.0 | 4.6 | 8.4 | 11.52 | 0.53 | 18.8 |
| 58 | 1.25 | 4 | 26.4 | 4.8 | 5.5 | 15.59 | 1.57 | 32.8 |
| 98 | 1.25 | 4 | 40.4 | 4.9 | 8.2 | 16.42 | 2.50 | 52.4 |
| 58 | 20 | 4 | 26.1 | 5.6 | 4.7 | 17.85 | 0.12 | 4.2 |
| 98 | 20 | 4 | 36.0 | 6.0 | 6.0 | 13.11 | 0.25 | 8.4 |
| 58 | 10.625 | 2 | 49.2 | 5.1 | 9.7 | 15.81 | 1.23 | 21.4 |
| 98 | 10.625 | 2 | 51.1 | 7.0 | 7.3 | 16.91 | 3.51 | 62.5 |
| 78 | 1.25 | 2 | 33.3 | 5.6 | 6.0 | 11.65 | 2.01 | 41.9 |
| 78 | 20 | 2 | 45.0 | 6.9 | 6.6 | 14.16 | 0.25 | 8.4 |
| 78 | 10.625 | 0.5 | 27.1 | 4.5 | 6.1 | 10.56 | 0.75 | 13.4 |
| 78 | 10.625 | 4 | 18.0 | 5.2 | 3.6 | 11.52 | 0.62 | 11.0 |
| 78 | 10.625 | 2 | 32.8 | 5.6 | 5.9 | 15.59 | 1.05 | 18.7 |
| 78 | 10.625 | 2 | 32.5 | 5.5 | 5.9 | 16.42 | 1.23 | 21.9 |
| 78 | 10.625 | 2 | 33.2 | 5.6 | 6.0 | 17.85 | 1.15 | 20.5 |


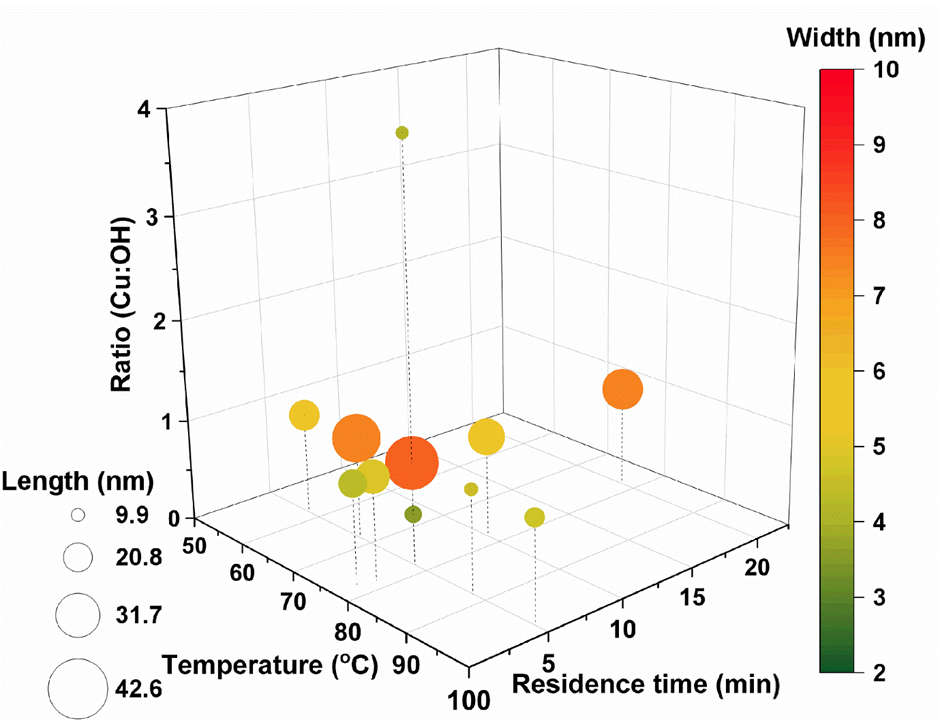


**Figure S9.** OFAT plot showing the effect of the conditions on the length and width of the CuO nanoparticles.


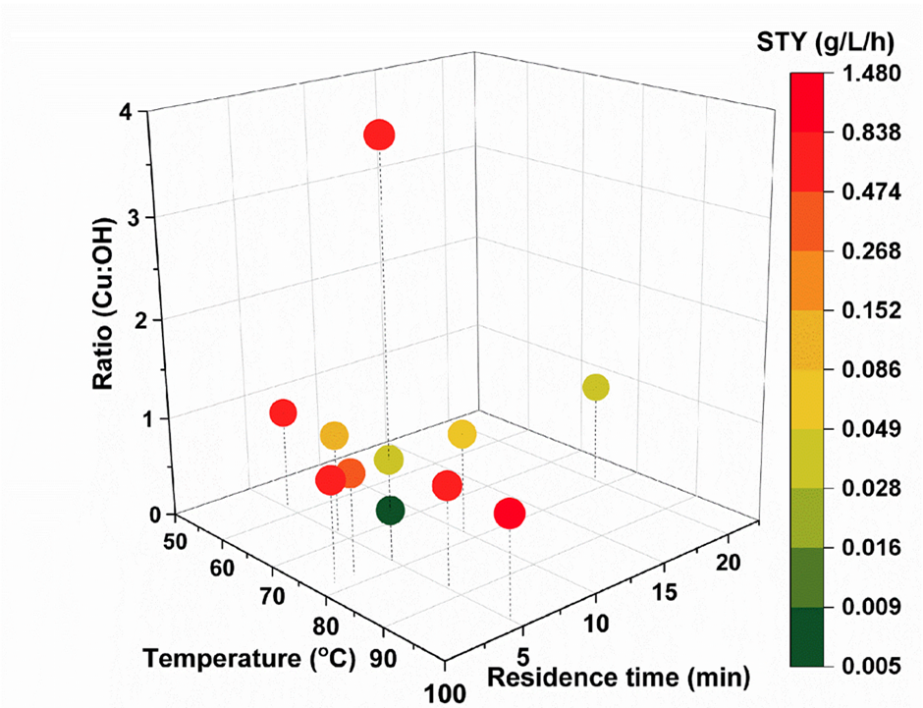


**Figure S10.** OFAT plot showing the space time yield (STY) of CuO nanoparticles synthesised in the microfluidic device under various conditions.

**
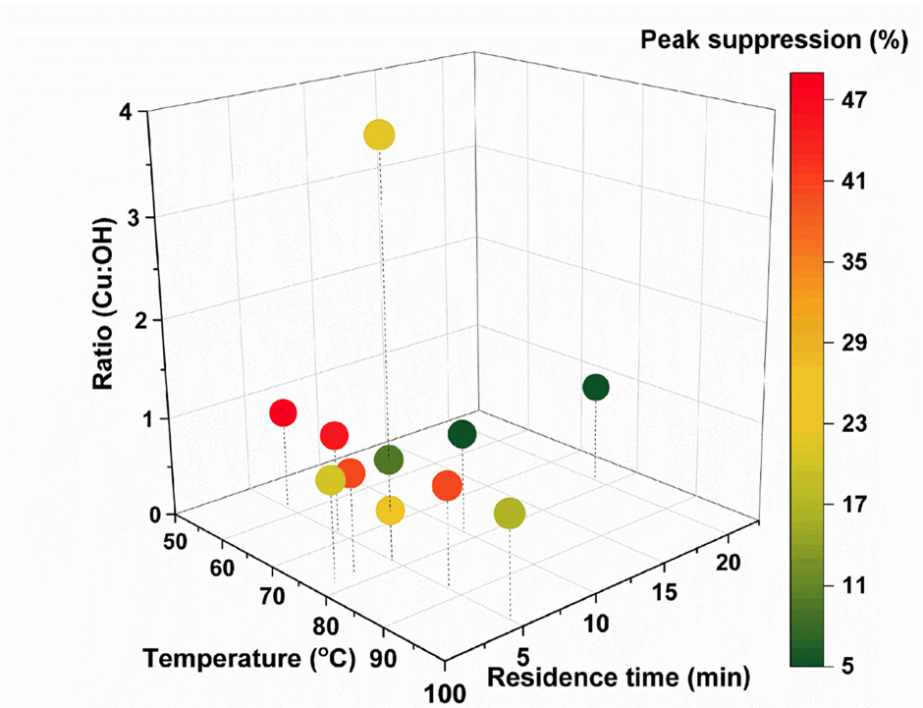
**

**Figure S11.** OFAT plot showing the peak suppression of the CuO nanoparticles synthesised in flow. The peak suppression % was observed via the RCV trace in the artificial biomembrane sensor. Longer residence times (>5 minutes) at 70^o^C produced low peak suppression % (<15%).


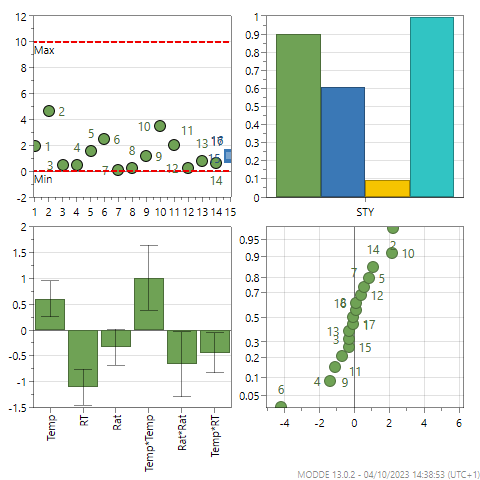


**Figure S12.** Overall summary of the DoE CCF for the space time yield, which shows a high reproducibility in the peak suppression for the replicates (top left), summary of the fit (green = R2, dark blue = Q2, yellow = model validity & light blue = reproducibility) (top right), the coefficient plot which provide graphical presentation of the significance of the model terms (bottom left) and the residual normality which shows that the residuals are normally distributed, and the points on the probability plot follow close to a straight line.

**
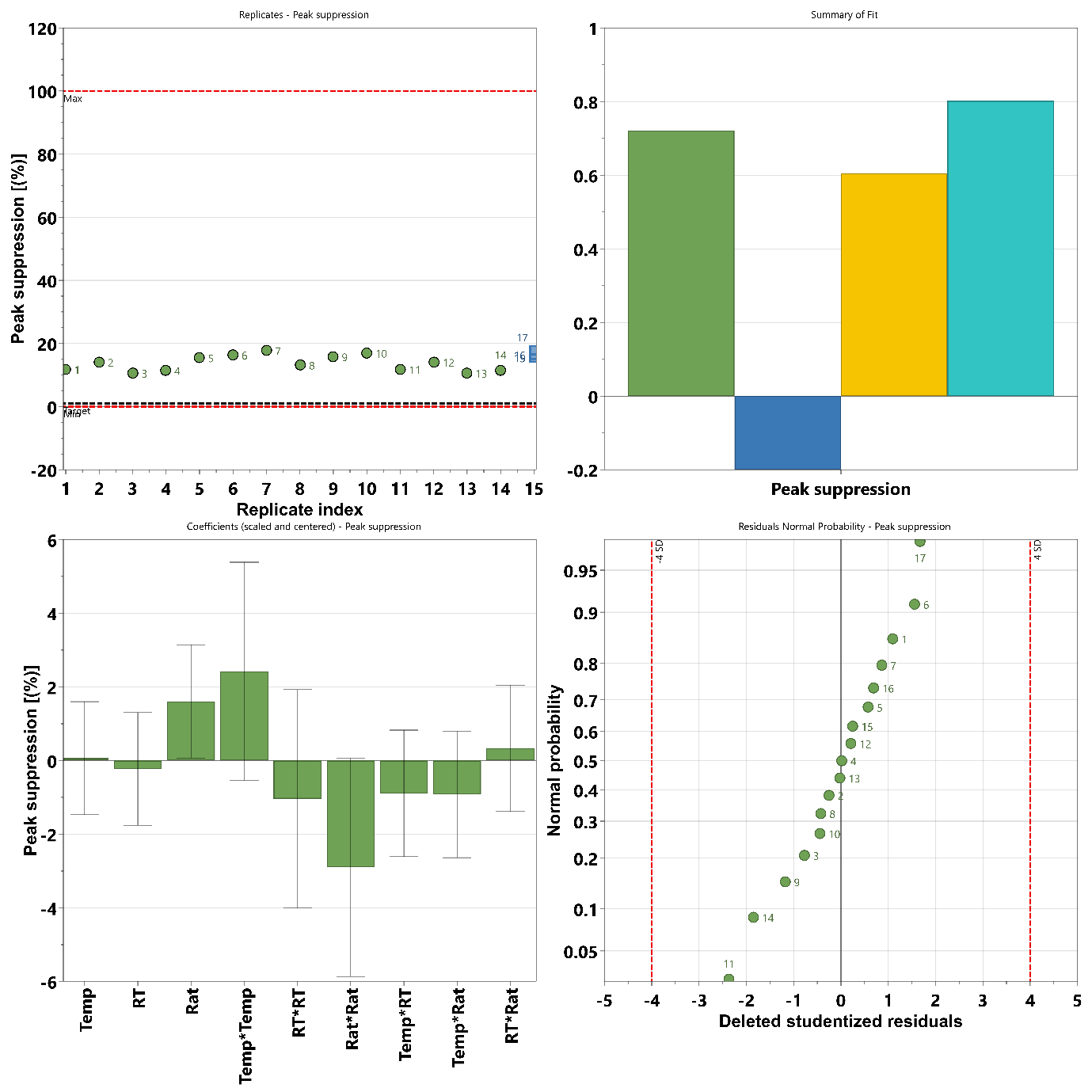
**

**Figure S13.** Overall summary of the DoE CCF for the peak suppression, which shows a high reproducibility in the peak suppression for the replicates (top left), summary of the fit (green = R2, dark blue = Q2, yellow = model validity & light blue = reproducibility) (top right), the coefficient plot which provide graphical presentation of the significance of the model terms (bottom left) and the residual normality which shows that the residuals are normally distributed, and the points on the probability plot follow close to a straight line.

**
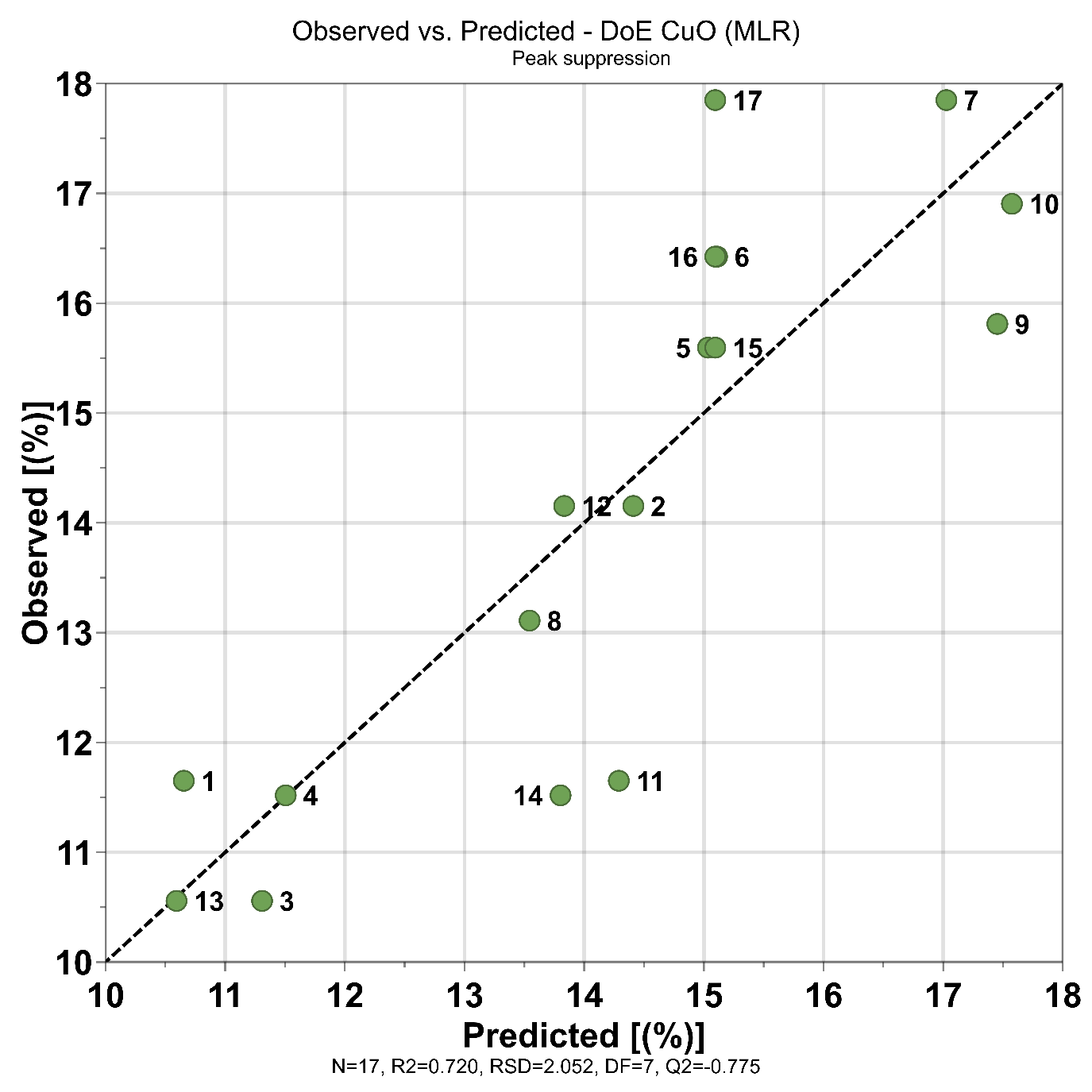
**

**Figure S14.** Scatter plot showing the observed vs. predicted peak suppression of the 17 experiments in the CCF DoE.

**
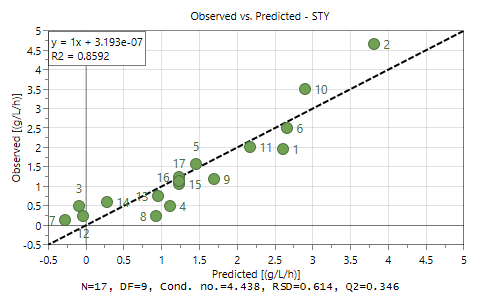
**

**Figure S15.** Scatter plot showing the observed vs. predicted space time yield (STY) of the 17 experiments in the CCF DoE.
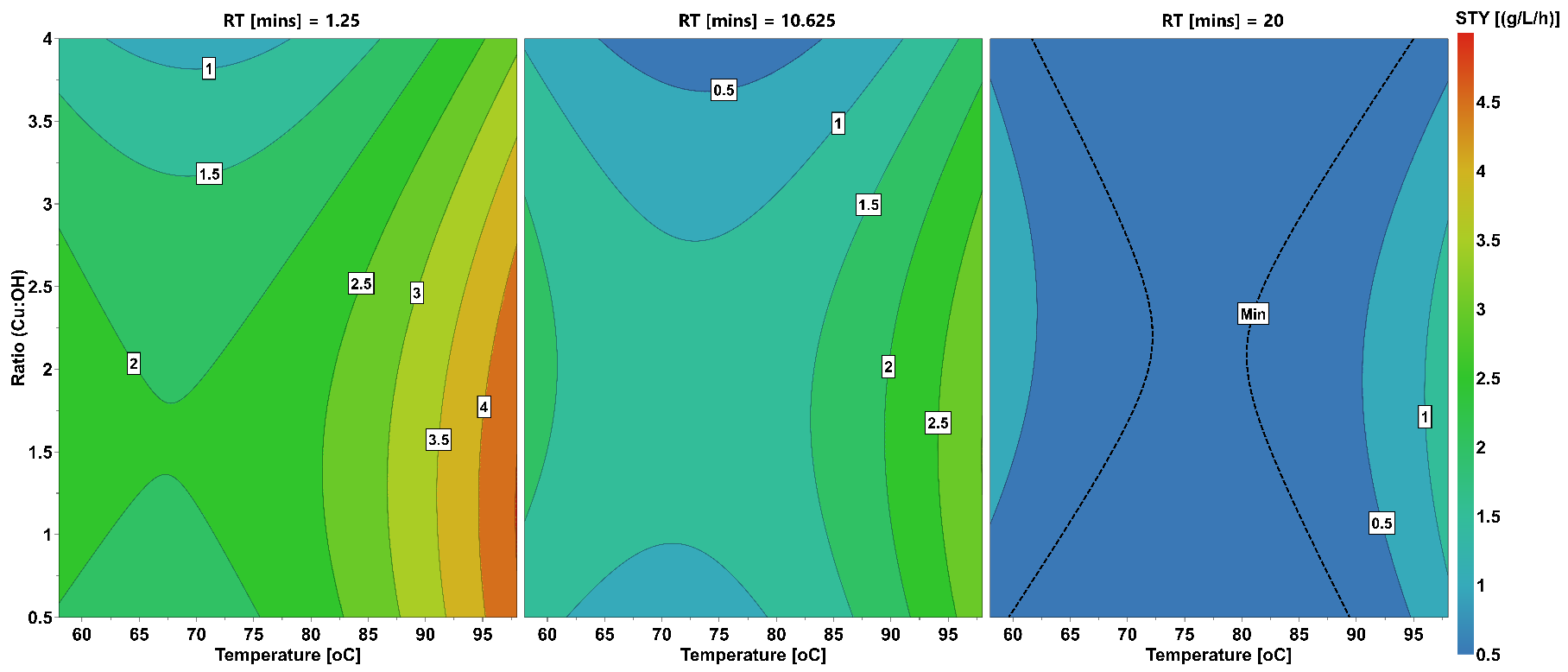


**Figure S16.** DoE CCF 4D contour plot showing the effect of the experimental conditions on the space time yield**.** Observing at short residence times and high temperatures we produce the most amount of product.


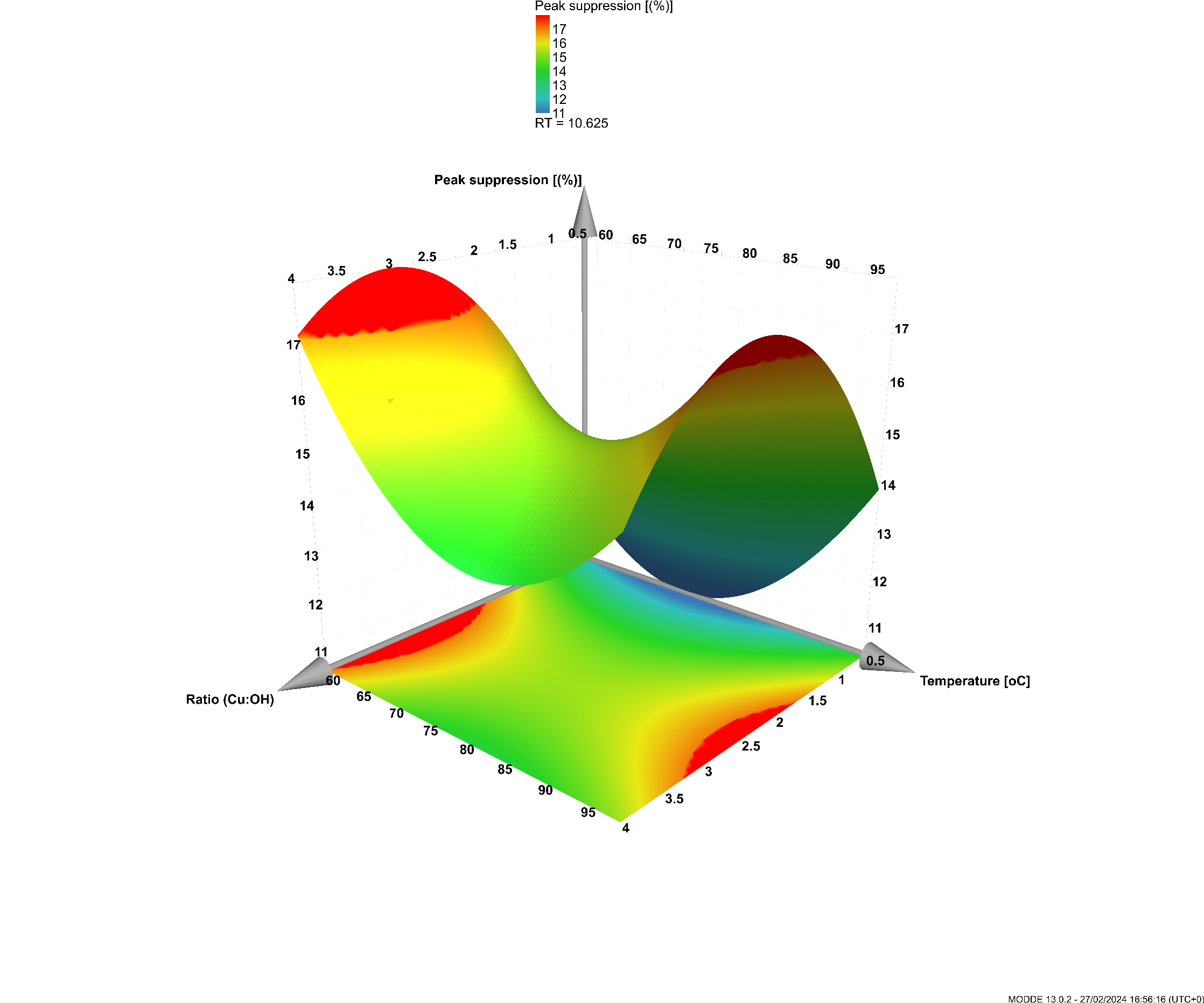


**Figure S17.** Surface plot showing the alteration of temperature and ratio on the peak suppression at a residence time of 10.63 minutes.


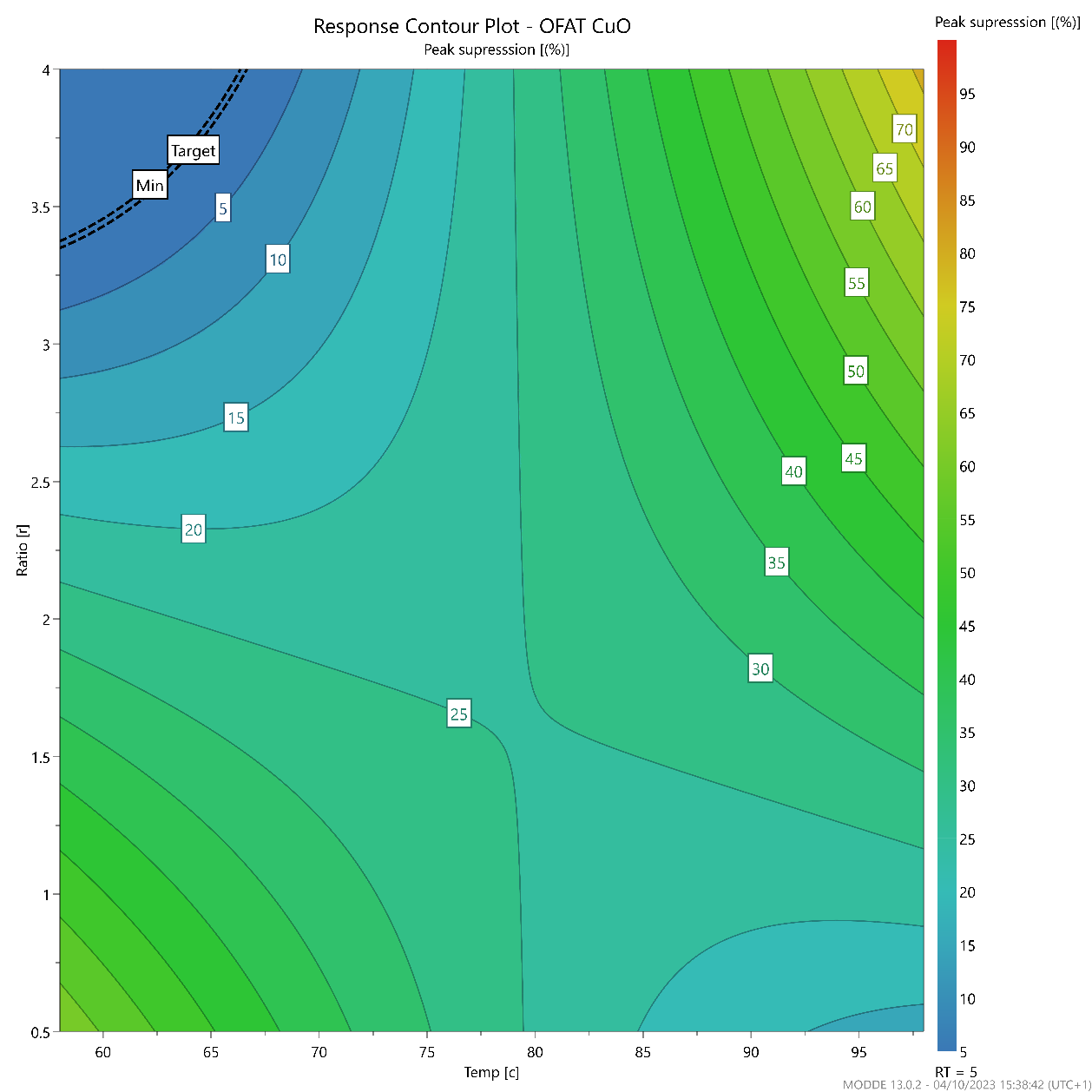


**Figure S18.** Contour plot showing the effect of temperature and ratio of copper precursor on peak suppression in the OFAT at a residence time of 5 minutes.


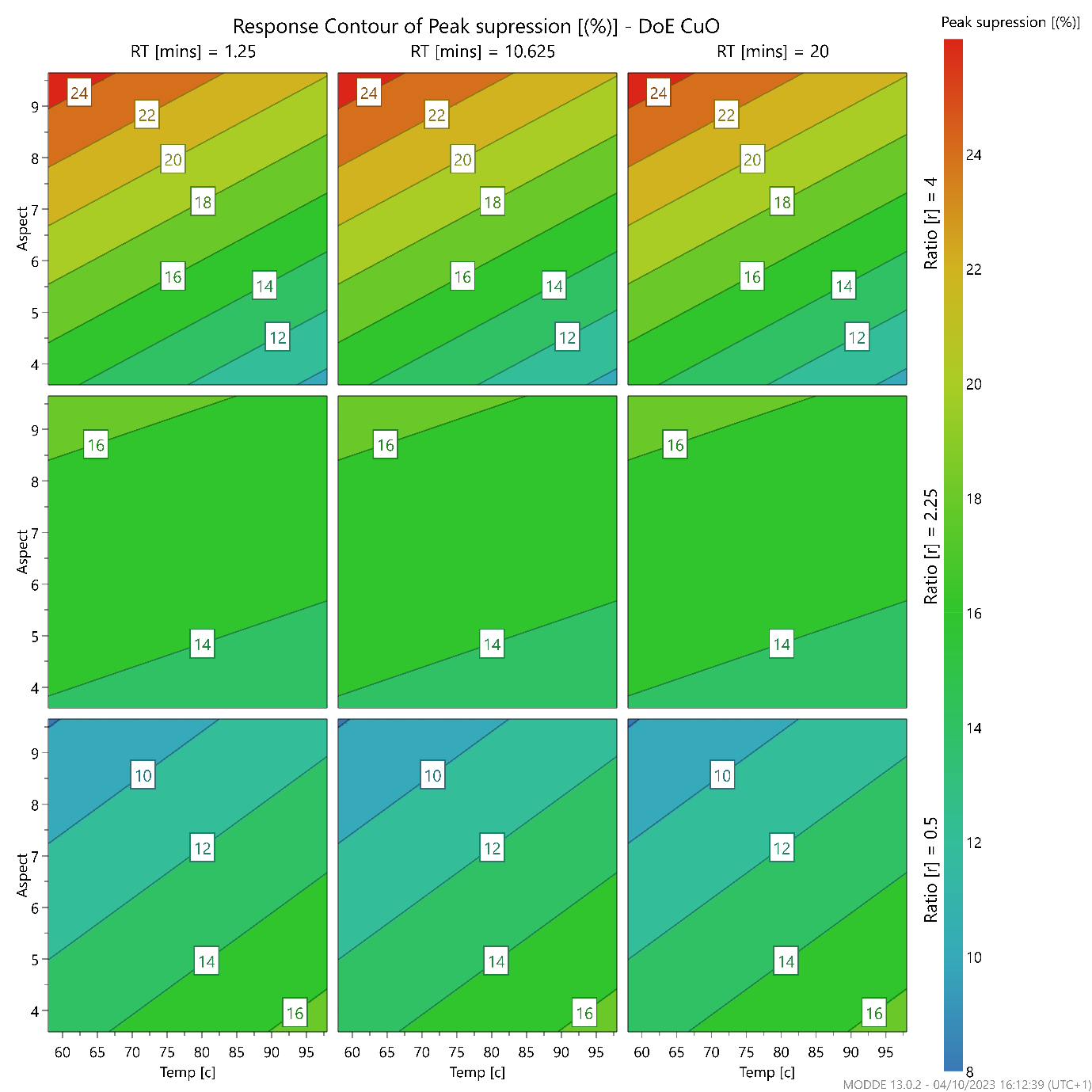


**Figure S19.** 4D contour plot showing the effect of temperature, residence time, aspect ratio and ratio of copper precursor on peak suppression in the DoE. Observing that higher aspect ratios at higher concentrations of Cu to reductant gave a strong peak suppression signal.

**
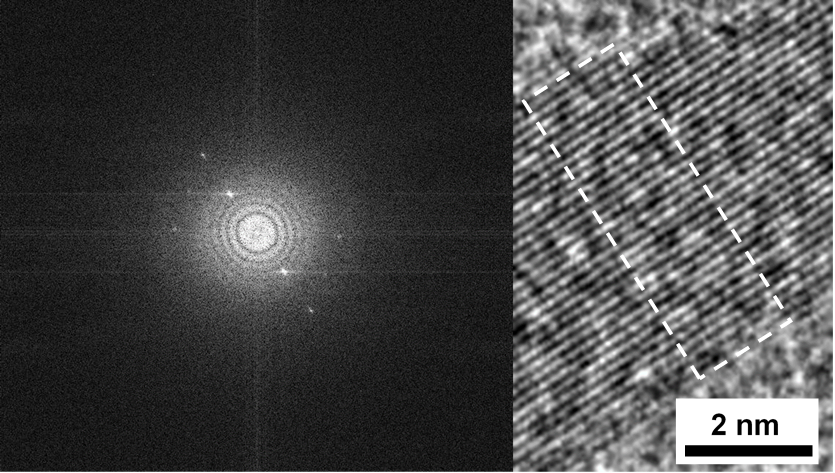
**

**Figure S20.** TEM image of the CuO nanoparticle showing the lattices of the material. The box is the measurement area for the line profile seen in SI figure 21 & is corresponding to Fig 1Dii in the manuscript.


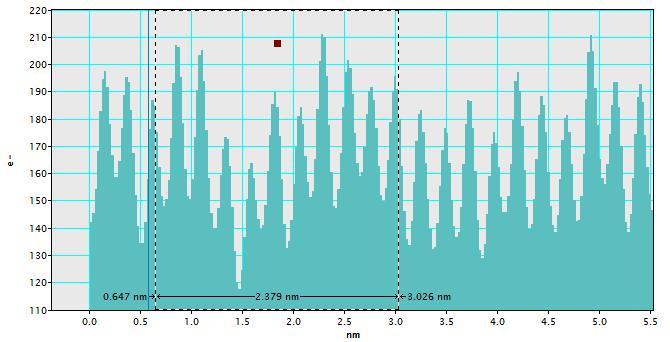


**Figure S21.** – Corresponding line profile of the e^-^ count against space for the CuO needle in the white dashed lined box drawn in Fig 1Dii & SI Fig 20.


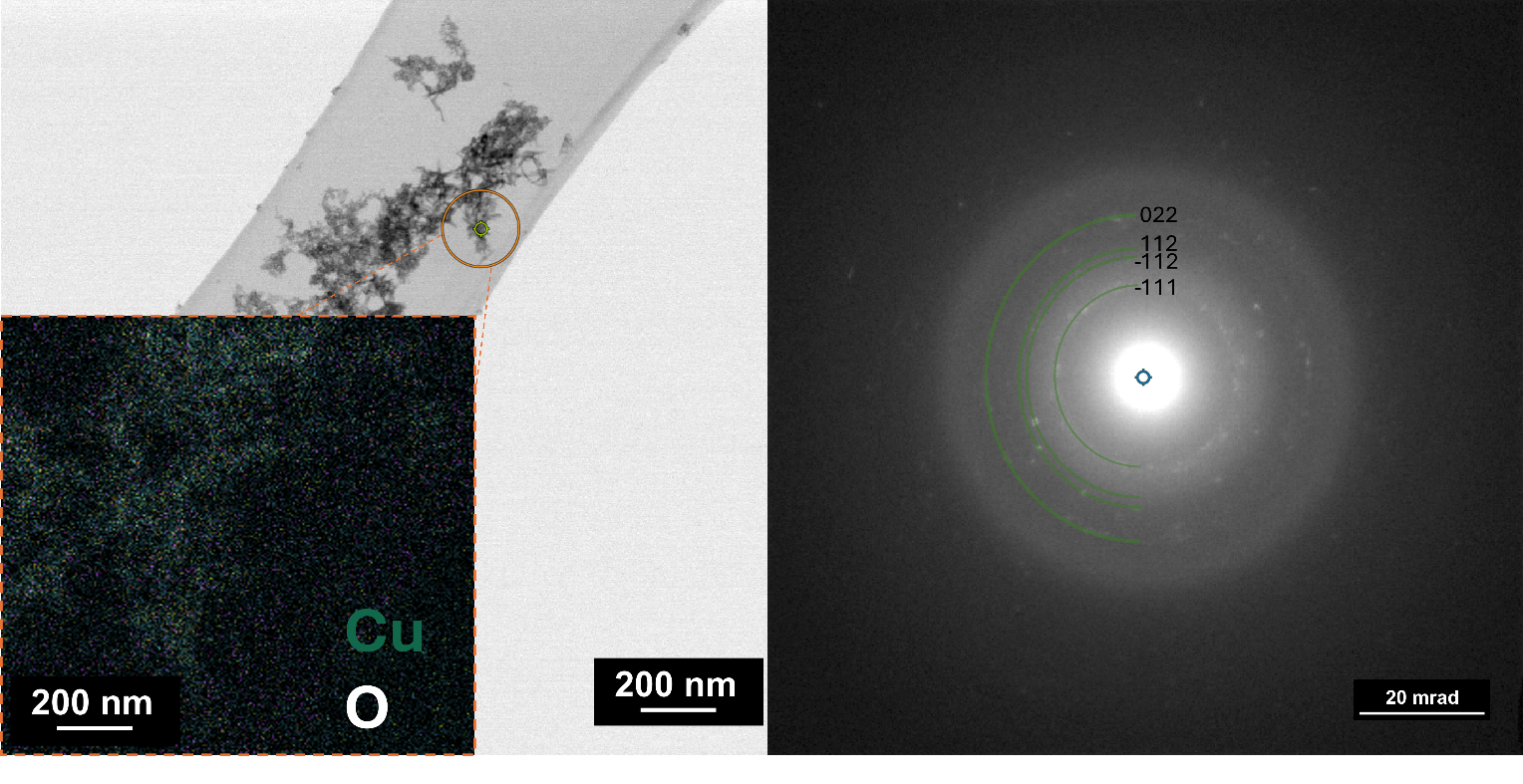


**Figure S22**. SAED of the CuO nanoparticles (right) from the circled region in the TEM image (left) with a composite EDX map inset.


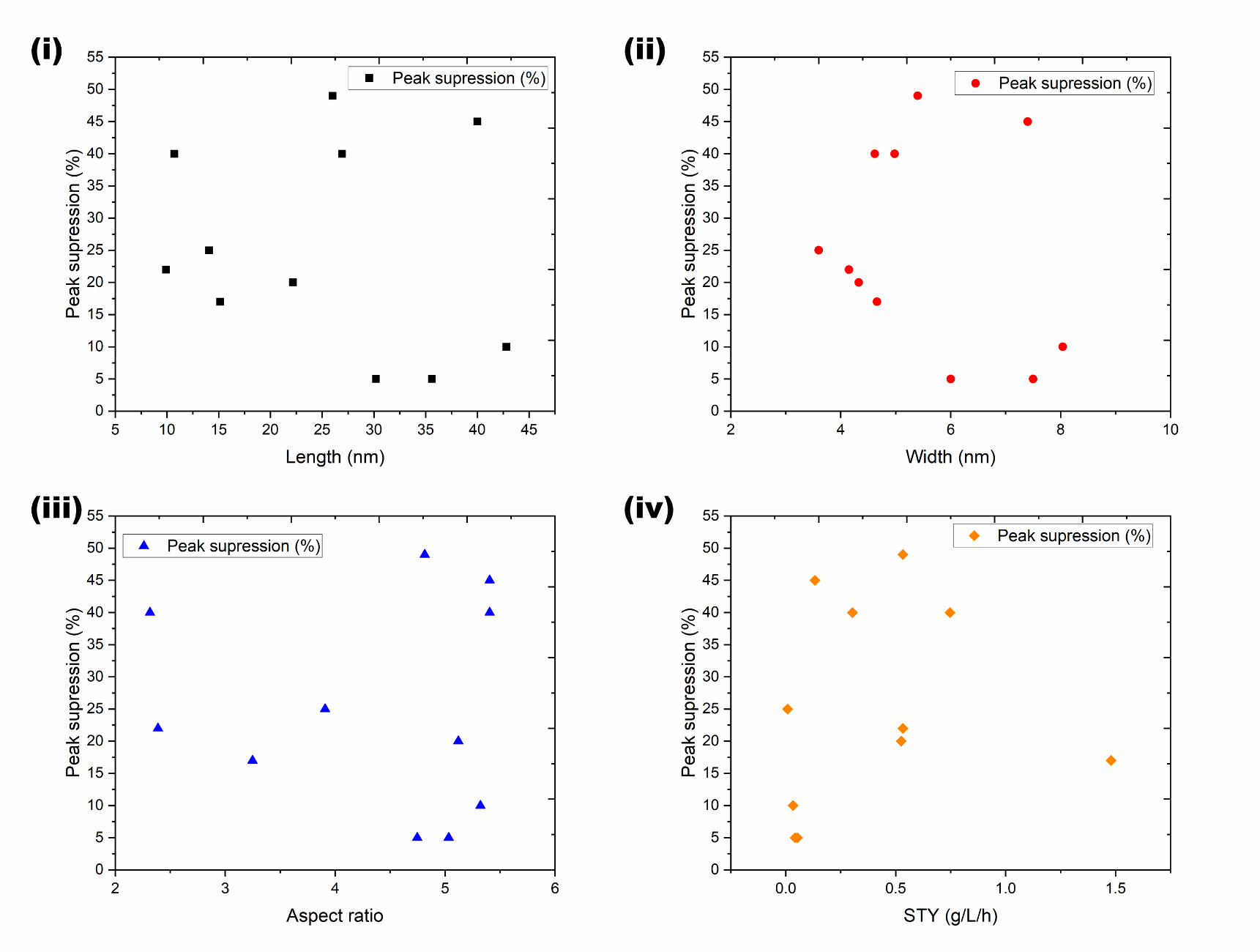


**Figure S23**. One factor at a time (OFAT) data showing the effect of length (i), width (ii), aspect ratio (iii), and space time yield (iv) on peak suppression (%).


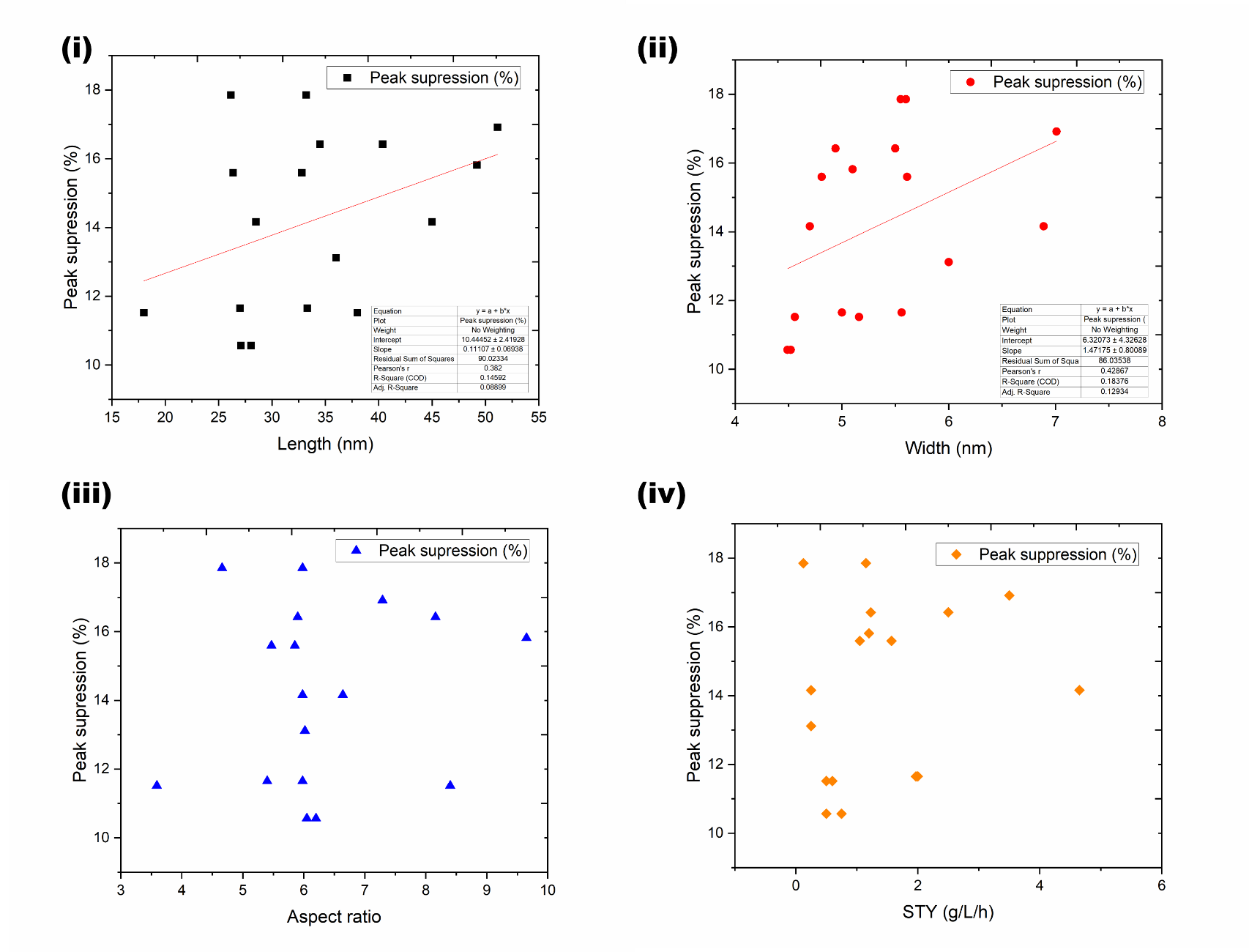


**Figure S24**. Design of experiments (DoE) data showing the effect of length (i), width (ii), aspect ratio (iii), and space time yield (iv) on peak suppression (%).


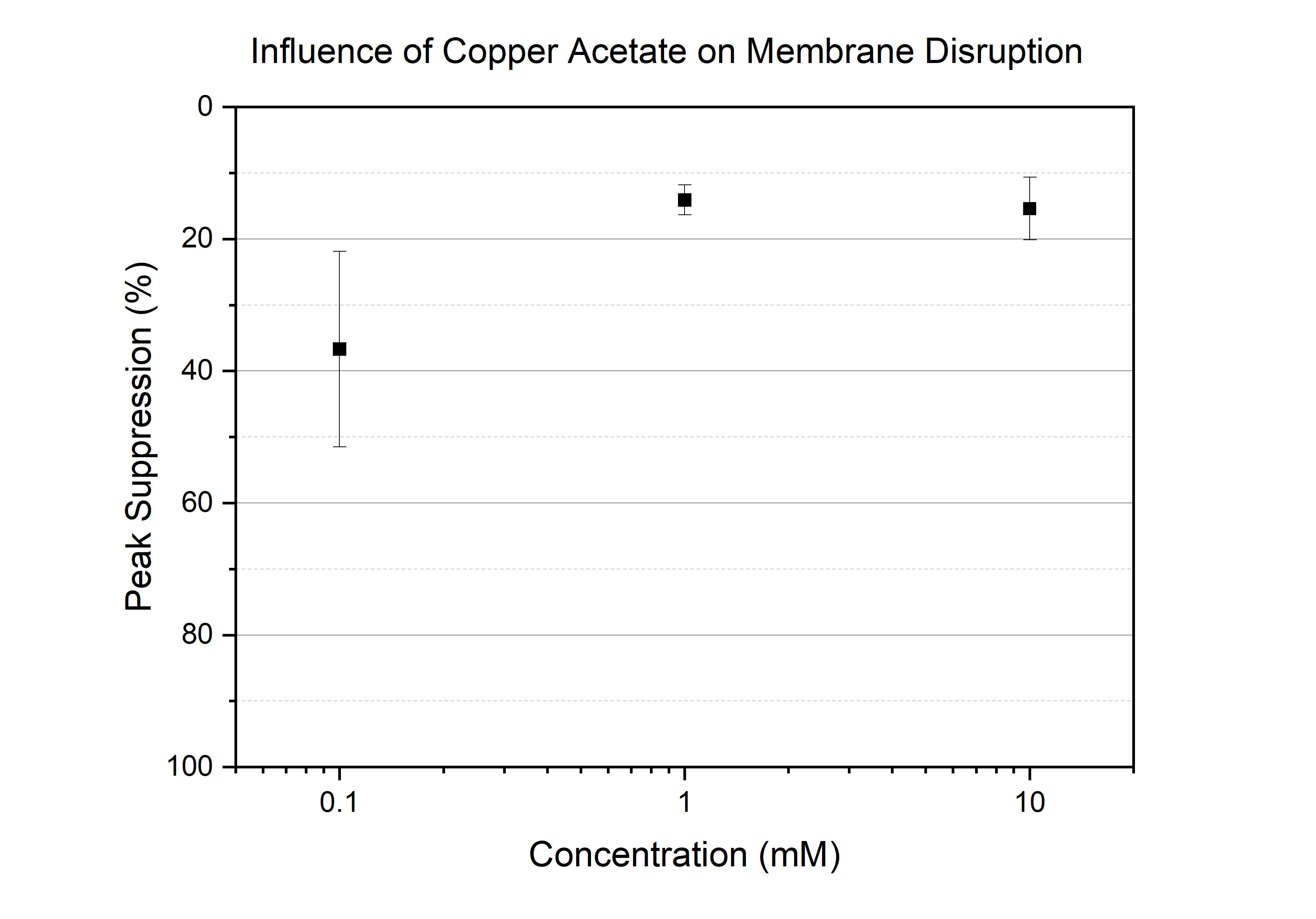


**Figure S25.** Influence of the copper acetate precursor on the peak suppression in the artificial biomembrane sensor.

**References:**

1. J. Owen, M. Kuznecovs, R. Bhamji, N. William, N. Domenech-Garcia, M. Hesler, T. Knoll, Y. Kohl, A. Nelson, N. Kapur, Rev. Sci. Instrum. 2020, 91, 025002
2. Zhang, S., Nelson, A. and Beales, P.A.J.L. Freezing or wrapping: the role of particle size in the mechanism of nanoparticle–biomembrane interaction. 2012, 28(35), pp.12831-12837.
